# Developing contraceptive services for immigrant women postpartum – a case study of a quality improvement collaborative in Sweden

**DOI:** 10.1186/s12913-022-07965-9

**Published:** 2022-04-26

**Authors:** Helena Kilander, Maja Weinryb, Malin Vikström, Kerstin Petersson, Elin C. Larsson

**Affiliations:** 1 Jönköping Academy for Improvement of Health and Welfare, School of Health and Welfare Jönköping University, Jönköping, Sweden; 2 Department of Women’s and Children’s Health, Karolinska Institutet, Solna, Sweden; 3 Division of Nursing Sciences and Reproductive Health, Department of Health, Medicine and Caring Sciences and Department of Obstetrics and Gynaecology, Region Jönköping County, Linköping University, Linköping, Sweden; 4 Health Care Services, Stockholm Region, Stockholm, Sweden; 5 Maternal Healthcare Unit, Stockholm South General Hospital, The Health and Medical Care Administration, Region Stockholm County, Stockholm, Sweden; 6 Department of Clinical Sciences, Obstetrics and Gynaecology, Umeå University, Umeå, Sweden; 7 Department of Global Public Health, Karolinska Institutet, Widerströmska huset, floor 3, Tomtebodavägen 18A, SE-171 77 Stockholm, Sweden; 8 Department of Women’s and Children’s Health, Uppsala University, Uppsala, Sweden

**Keywords:** Contraception, Counselling, Coproduction, Family planning, Maternal health care, Postpartum, System performance, Quality improvement

## Abstract

**Background:**

Immigrant women use less effective contraceptive methods and have a higher risk of unintended pregnancies. Maternal health care services offer a central opportunity to strengthen contraceptive services, especially among immigrants. This study aimed to evaluate a Quality Improvement Collaborative QIC. Its objective was to improve contraceptive services for immigrant women postpartum, through health care professionals’ (HCPs) counselling and a more effective choice of contraceptive methods.

**Methods:**

The pilot study was designed as an organisational case study including both qualitative and quantitative data collection and analysis. Midwives at three maternal health clinics (MHCs) in Stockholm, Sweden participated in a QIC during 2018–2019. In addition, two recently pregnant women and a couple contributed user feedback. Data on women’s choice of contraceptive method at the postpartum visit were registered in the Swedish Pregnancy Register over 1 year.

**Results:**

The participating midwives decided that increasing the proportion of immigrant women choosing a more effective contraceptive method postpartum would be the goal of the QIC. Evidence-based changes in contraceptive services, supported by user feedback, were tested in clinical practice during three action periods. During the QIC, the proportion of women choosing a more effective contraceptive method postpartum increased at an early stage of the QIC. Among immigrant women, the choice of a more effective contraception increased from 30 to 47% during the study period. Midwives reported that their counselling skills had developed due to participation in the QIC, and they found using a register beneficial for evaluating women’s choice of contraceptive methods.

**Conclusions:**

The QIC, supported by a register and user feedback, helped midwives to improve their contraceptive services during the pregnancy and postpartum periods. Immigrant women’s choice of a more effective contraceptive method postpartum increased during the QIC. This implies that a QIC could increase the choice of a more effective contraception of postpartum contraception among immigrants.

## Background

Contraception has a wide range of benefits for women’s sexual and reproductive health and rights (SRHR). Effective contraceptive methods such as long-acting reversible contraception (LARC) e.g. intrauterine device, and short-acting reversible contraception (SARC) e.g. the pill, are associated with a lower risk of unintended pregnancies (UPs), abortions as well as pregnancy-related adverse events [1–3].

Pregnancy and the postpartum period offer a key opportunity to facilitate women’s choice of more effective contraception [1, 4] since fertility returns quickly after childbirth for women who are not breastfeeding [4]. Double counselling sessions, i.e. counselling during pregnancy *and* the postpartum period increase contraceptive use after giving birth as compared to counselling *exclusively* postpartum [5, 6]. Double sessions seem feasible for and are accepted by women [7] and health care professionals (HCPs) alike [8]. Furthermore, person-centred approaches and the use of visual tools illustrating the effectiveness of different contraceptive methods, facilitate women’s choice of methods [9–11]. However, little is known of how these interventions enable immigrant women’s choice of contraception. Immigrant women in high-income countries report lower use of effective contraception, and higher rates of UPs and abortions compared to native-born women [12, 13]. Possible explanations for these differences are negative experiences of contraceptive counselling postpartum [7, 14, 15] and limited support from the partner [16], as well as health care providers’ difficulties adapting care to persons with low health literacy, cultural differences, and language barriers [17, 18]. In Sweden, about 30% of women giving birth are immigrants born outside of Sweden. The immigrant population in Sweden is heterogeneous, with the most common countries of birth for persons born outside of Europe being: Syria, Iraq, Iran and Somalia at the time of the study [19].

In Sweden, few women choose more effective contraceptive methods within 12 weeks after giving birth [20]. Repeat abortions increase during the childbearing age [21]. Quality Improvement Collaboratives (QICs) can support HCPs in improving health care and contraceptive services in clinical practice [10, 22–24] and can increase user involvement [22, 25, 26].

Swedish National Quality Registers have successfully been used to evaluate QICs in health care [23, 24, 27], but have never been used in contraceptive services. This sentence belongs to, should be directly after user involvement.

## Methods

This pilot study aimed to evaluate a QIC. Its objective was to improve contraceptive services for immigrant women postpartum, through health care professionals’ (HCPs) counselling and resulting in women choosing a more effective contraceptive method. The Swedish Pregnancy Register was used to register data on the choice of contraceptive method. This study was reported according to the SQUIRE guidelines [28].

### Setting

This study was carried out at three maternal health clinics (MHCs), the main organisations responsible for postpartum care in Sweden. One or more postpartum visits, including contraceptive services, should be offered to all women within 16 weeks after giving birth [29]. Midwives prescribe and administer the majority of all contraceptive methods, including LARC [30].

### Study design

The QIC took place in 2018–2019, within the regular health care setting at three MHCs in two urban municipalities in the outer regions of Stockholm County. The municipalities differed in size and percentage of immigrant inhabitants. The study was designed as an organisational case study [31] describing a QIC, involving qualitative and quantitative data collection methods. The QIC was based on the Breakthrough model [32] and was inspired by a previous study using QIC for the improvement of contraceptive services [33].

### Design of Quality Improvement Collaborative (QIC)

Three MHCs were invited to participate in the QIC. They agreed to register information about women’s choice of contraceptive method postpartum in the Swedish Pregnancy Register (SPR) during the period and to take part in learning seminars (LSs) during the QIC. There was no national data regarding women’s choice of postpartum contraception, as such information was not currently collected nationally in Sweden.

**Fig. 1:**
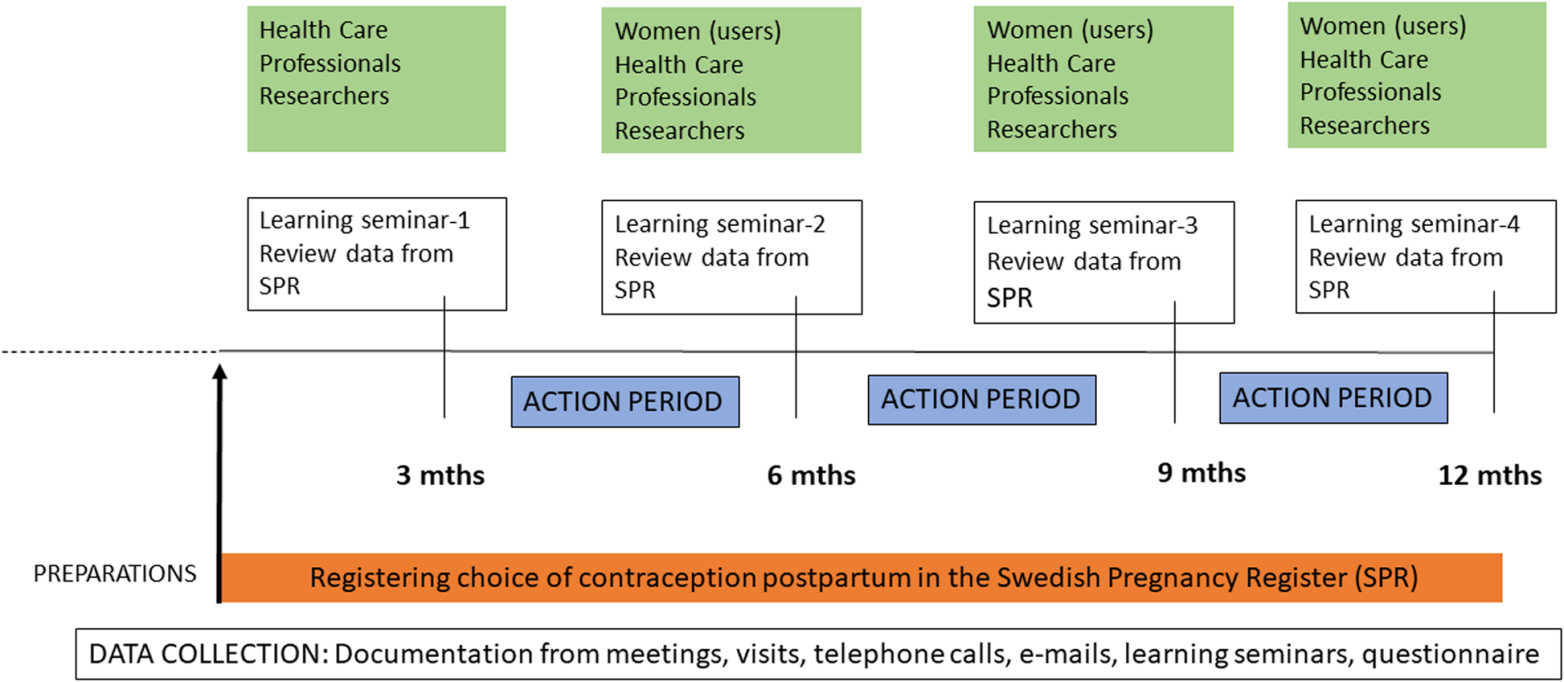
Timeline for the quality improvement collaborative, user involvement and the case study data collection 2018-2019

Preparations for data collection in the SPR started during spring 2018 and involved meetings with the project leader, researchers, midwives and heads of the MHCs (Fig. 1). The first learning seminar was held in December 2018, 3 months after the MHCs had first started registering contraceptive methods in the SPR, and the last one was held in October 2019. Data were continuously registered and extracted from the SPR to review women’s choice of contraceptive methods during the whole QIC period (Fig. 1).

All the authors supported and ran the QIC according to the Breakthrough model. MV served as a coordinator in her position as maternal care coordinator midwife for maternal health care in the Stockholm Region. KP supported the MHCs with statistics from the SPR. HK, EL and MW planned and conducted the learning seminars. Two of the MHCs participated in all four LSs, whereas one MHC participated in the last three LSs. In total, 10–13 midwives participated in each LS.

The QIC involved four LSs, encompassing lectures, discussions, feedback and analysis of data from the SPR as well as time for sharing experiences. The midwives were introduced to a framework for improvement in clinical practice using the Driver Diagram and Plan-Do-Study-Act cycles [34] (Table 1). Contraceptive services in this study were defined as counselling as well as prescription and administration of contraceptive methods.

**Table 1:** Programme for Learning Seminars (LS) during Quality Improvement Collaborative (QIC) 2018–2019

Learning Seminars (LS)	Themes for the seminar	Activities at the seminar Discussions/teamwork	Homework and activities after the seminar
**LS 1** (Dec 2018)	*Why do we need to improve contraceptive services?* *Update relevant research from the field regarding:* Risk-factors for unintended pregnancy Best practice contraceptive counselling postpartum *Local data from the SPR regarding women’s choice of contraceptive methods postpartum* *Improvement in health care and theory.* Introduction of driver diagram Introduction to the tool Plan-Do-Study-Act How to work in a team in a QIC	Identify obstacles in; -providing contraceptives services postpartum -registering women’s choice of contraceptive method postpartum in the SPR Introduction to visual tool illustrating the effectiveness of contraceptive methods Discuss and set goals for the QIC: What do we want to accomplish?	Continue to identify possible obstacles and possibilities at each MHC Continue to register women’s choice of contraceptives postpartum in the SPR Test visual tool illustrating the effectiveness of different contraceptive methods Test offer information about contraceptive methods during gestational weeks 36–38 Test book the postpartum visit during pregnancy.
**LS 2** (March 2019)	*Analyse results in SPR.* *RLP, a tool for person-centred counselling* *Midwives’ experiences of improvement activities* *Clarify the goals of the work.* *What changes are we planning to make?*	Share experiences of registering women’s choice of contraceptive method in the SPR Reflect on how to use RLP in the conversation about contraception Share experiences of improvement activities Choose future testing areas	Continue to register women’s choice of contraceptives postpartum Continue using the visual tool Continue offering information about contraceptive methods and offer prescriptions during gestational weeks 36–38 Test book appointments for contraceptive services postpartum Test to develop a stock of LARC
**LS 3** (June 2019)	*Analyse results regarding women’s choice of contraceptive method in the SPR* *Lecture in best practice contraceptive methods postpartum, including natural family planning* *User feedback*: *Immigrant women’s and Swedish-born women’s views on teams’ improvement activities* *Midwives’ experiences of improvement activities*	Share experiences of register women’s choice of contraceptive method postpartum Share experiences of improvement activities Reflect on users’ views in the QIC	Continue to register women’s choice of contraceptives postpartum Continue using the visual tool Continue offering information about contraceptive methods and prescription during gestational weeks 36–38 Continue to book appointments for contraceptive services postpartum Maintain the stock of LARC
**LS 4** (Oct 2019)	*Analyse the results so far from the SPR* *User feedback: Immigrant women’s and Swedish-born women’s views on teams’ actions* *Future work*	Reflect on users’ views in the QIC How to keep on reaching the goal? How can we create sustainability?	Continue to register women’s choice of contraceptives postpartum Continue using the visual tool Continue offering information about Contraceptive methods during gestational weeks 36–38 Continue to book appointments for contraceptive counselling postpartum Maintain the stock of LARC

*LS* Learning Seminar, *LARC* Long-Acting Reversible Contraception, *RLP* Reproductive Life Planning, *SPR* Swedish Pregnancy Register, *MHC* Maternal Health Clinic, *QIC* Quality Improvement Collaborative

**Fig. 2:**
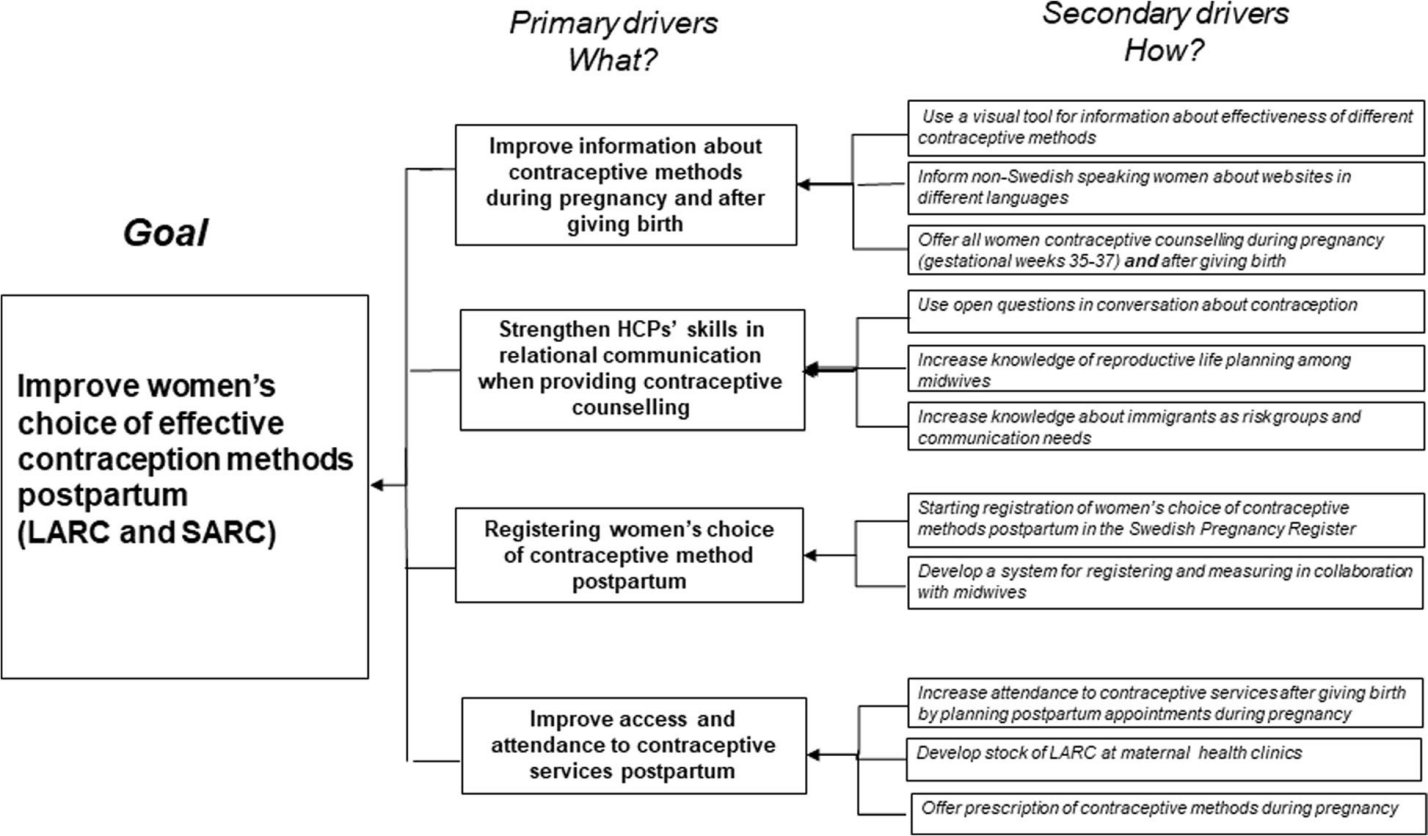
The quality improvement collaborative based on the Driver diagram [34], outlining the four main areas for evidence-based changes

Four evidence-based areas of change regarding contraceptive services (primary drivers) were presented to the midwives, based on previous research [26, 33]. These four drivers were expected to improve women’s choice of more effective contraceptive methods postpartum (the goal). As a means to achieve the four primary drivers, the MHCs chose improvement activities (secondary drivers), adapted to the clinical setting of each MHC (Fig. 2). The improvement activities were actively tested in clinical practice between the LSs, i.e. during the action periods (Fig. 1, 2).

The QIC was supported by user feedback, captured through interviews held by authors MV & HK. Two women (one immigrant and one Swedish-born), as well as one immigrant couple, shared their experience of contraceptive services postpartum, and their opinions about the chosen improvement activities. De-identified data from the user perspective were reported back to the MHCs during LSs.

### Qualitative data collection and analysis

Qualitative data consisted of meeting notes from researcher visits to the MHCs, email correspondence, notes taken during telephone calls, verbatim transcripts from audio recordings during the LS, and an anonymous questionnaire from the final LS. Data were analysed using content analysis on a manifest level by the three authors (HK, EL and MW) [35]. All transcripts were read repeatedly by three of the authors (listed above). Data regarding the QIC and midwives’ experiences of developing contraceptive services postpartum were highlighted, coded and grouped into different categories based on the driver diagram. Quotations were presented in the manuscript to illustrate the findings. Coding and categorisation were discussed among the three researchers as a form of validation and triangulation [35].

### Quantitative data collection and analysis

Data on the choice of contraception as well as background characteristics for women attending postpartum visits between September 2018–October 2019 were registered and collected from the SPR during the study period. No data were collected from the women themselves. More effective contraception was defined as SARC (contraceptive pill, patch, ring and injectables) and LARC (subdermal implant and intrauterine devices [36]). Less effective methods were defined as methods with pearl index < 9, such as barrier methods, withdrawal, natural family planning or choice of no method at all [30]. The proportions of women choosing a more effective contraceptive method are presented for each of the time points of the LSs and stratified by all women and immigrant women. Chi-square statistics were used to analyze the increase in the choice of more effective contraceptive methods between LS 1 and 4, presented in percentages and *p*-values.

## Ethical considerations

Ethical approval for undertaking this study was obtained from the regional ethics committee in Stockholm ref. 2017/1312–31/5 and an additional application, ref. 2108/1241–32. The project was performed in accordance with the Declaration of Helsinki. The register data used from the Swedish Pregnancy Register (i.e. a quality register) were used without any identifiers for individual women. Women are informed that data is collected in the SPR and that they could decline or withdraw their participation at any time. The head of the clinics gave written informed consent to participate in the study. The two participating women and one couple who gave feedback on their experiences gave their written informed consent to participate in the study.

## Results

### Activities decided upon for the learning seminars and action periods

In LS 1, the midwives jointly agreed on a common goal for the QIC: to increase immigrant women’s choice of more effective contraceptive methods postpartum. In the subsequent LSs, the midwives took part in analysing data from the SPR and reflected on their progress towards reaching the goal. They were guided to plan improvement activities (secondary drivers) in their MHC according to the chosen primary drivers in the Driver Diagram (Fig. 2). By sharing experiences, the midwives inspired each other in choosing improvement activities. The midwives chose activities that were feasible and/or adapted to the clinical setting of their own MHC. Furthermore, they were also invited to suggest the content in forthcoming LSs to improve contraceptive services. (Table 1).

## Quantitative results

### Immigrant women’s choice of more effective contraceptive methods

More than half of the women visiting MHCs during the QIC were immigrants, of which 27% were born in the Middle East/North Africa (Table 2). During the QIC, the proportion of all women choosing a more effective contraceptive method postpartum increased from 36 to 51%. The largest increase in the proportion of all women choosing a more effective method occurred between baseline/LS1 and LS2. Among immigrant women, the choice of more effective contraceptive method increased from 30 to 47% (Table 3).

**Table 2:** Background characteristics among the women visiting maternal health clinics (MHCs) and registered in the SPR during the QIC

Characteristic	Number of women (%)
**Clinic**	
MHC a	346 (61)
MHC b	129 (23)
MHC c	92 (16)
**Para**	
0	234 (41)
1	188 (33)
2	114 (20)
3+	31 (6)
**Level of education**	
No education/less than 9 years	6 (1)
Primary school (9 years)	34 (6)
Secondary school (12 years)	230 (41)
University	251 (44)
Missing	46 (8)
**Country of birth**	
Sweden	271 (48)
Middle-East/North-Africa	152 (27)
Other	144 (25)

*MHCs* Maternal Health Clinics, *SPR* Swedish Pregnancy Register, *QIC* Quality Improvement Collaborative

**Table 3:** Proportion of women choosing a more effective contraceptive method, by LS, all women (total) and immigrants

	Baseline/LS1 Pre-QIC 1st Sep-12th Dec 2018		LS2 (13th Dec 2018-5th March 2019)		LS3 (6th March-4th June 2019)		LS4 (5th June-31st Aug 2019)		Increase in more effective method in %		
	Less effective/ no method^b^ n (%)	More effective methods^a^ n (%)	Less effective/ No method^b^ n (%)	More effective methods^a^ n (%)	Less effective/ no method^b^ n (%)	More effective methods^a^ n (%)	Less effective/ no method^b^ n (%)	More effective methods^a^ n (%)	LS4 vs. LS1	LS3 vs. LS1	LS2 vs. LS1
**Immigrants**	51 (70)	**22 (30)**	30 (48)	**33 (52)**	34 (45)	**41 (55)**	45 (53)	**40 (47)**	**+ 16.9 (0.03)c**	**+ 24.5**	**+ 22.2**
**TOTAL**	98 (64)	**55 (36)**	49 (45)	**59 (55)**	66 (46)	**78 (54)**	80 (49)	**82 (51)**	**+ 14.7 (0.008)**^**c**^	**+ 18.2**	**+ 18.7**

^a^More effective contraception was defined as short-acting reversible contraception (SARC), including contraceptive pills, the combined hormonal contraceptive patch and ring, progestin only injectables and long-acting reversible contraception (LARC), including subdermal implant, intrauterine devices and levonorgestrel intrauterine systems. ^b^Less effective methods were defined as a choice of methods such as barrier methods, withdrawal, natural family planning or choice of no method at all

^c^Chi2-test was used to calculate *p*-values regarding the difference in choice of more effective contraceptive method between LS 4 and 1

*LS *learning seminars, *QIC *Quality improvement collaborative

## Qualitative results

During the first LS, midwives reported the experience that immigrant women chose less effective contraceptive methods such as withdrawal and condoms to a greater extent, which was confirmed by the quantitative data (Table 3).

### Development of contraceptive counselling – conveying information and relational aspects

Continuously during the QIC, midwives reported adopting improvement activities and thereby changing their approaches in contraceptive counselling for immigrant women. For example, they started to use the contraceptive effectiveness visual tool (Fig. 2, Table 1). Midwives expressed a need for better tools for counselling women not proficient in Swedish, encompassing printed information material as well as online resources in the women’s native languages. Midwives also articulated a concern regarding the depth of information in currently available translated materials.

Inspired by lectures during the LSs and supported by user feedback, midwives at all MHCs reported testing double contraceptive counselling sessions, i.e. counselling during pregnancy and postpartum (Table 1). All MHCs agreed to test this improvement activity in clinical practice and found it acceptable by both midwives and women.

*“I think it’s natural to talk about ovulation and the need for contraception postpartum …it feels better to initiate the topic at the end of pregnancy… I didn’t do it before; now it feels strange to exclude it …..the talk seems to prepare women …and I think it motivates attendance at the postpartum visit”* (Midwife MHC A)

Regarding the relational aspects of counselling, midwives described an increased use of open-ended questions after the QIC. Several midwives were positive about the experience of using a person-centred approach inspired by reproductive life planning, and had started asking questions such as “How many children would you like to have?, and “How long would you like to wait until you become pregnant again?”

Midwives reflected on the challenges of improving counselling for immigrant women and expressed limited knowledge on how to involve their partners. For example, midwives reflected on the fact that many men had never been educated and informed about contraceptive methods. Furthermore, they discussed the power dynamics, and how to empower the woman to freely choose a method, especially when she did not feel she had the agency to decide herself.

### Increasing attendance and access to contraceptive services postpartum

Midwives reported improvement activities aimed at increasing access and attendance to contraceptive services during postpartum visits. For example, the midwives started to routinely book appointments for postpartum visits during pregnancy, an improvement activity supported by user feedback. This proved important for women not proficient in Swedish. Midwives reported improvement activities manifested by prescribing and providing contraception during pregnancy, especially LARC.

*“I have offered and inserted more intrauterine devices and systems at the first visit six weeks postpartum, as women expressed the wish to resume sexual activity”* (Midwife MHC B)

### Experiences of registering women’s choice of contraceptive method

Midwives participated in the development of data collection in the SPR during postpartum visits. The midwives reported that registering the choice of contraceptive methods in the SPR was both feasible and helpful, by serving as a reminder to bring up the topic of contraception among other issues during postpartum visits. Several midwives thought these questions should be made permanent as they made them remember and focus on contraceptive counselling during the postpartum visit.

*“I did not systematically ask about women’s contraceptive needs… before we started to collect the data. It is a more natural part of the postpartum visit now compared to before”* (Midwife MHC C)

### Midwives’ experiences of participating in the QIC

Several midwives stated that involvement in the LSs had developed their skills in counselling and their knowledge of contraceptive methods, which made them more confident when meeting immigrant women and those sceptical towards hormonal contraception.

*“In consultations where women previously declined contraceptive methods… I have started to offer a conversation, which I didn’t before… I have also changed my approach to how I provide information and talk about contraception…. I include positive health effects nowadays and I believe that more women decide to use a method compared to before (this project)….”* (Midwife MHC A)

In the written evaluation after the QIC was finalised, almost all midwives reported that the tested improvement activities (secondary drivers, Fig. 2) would continue as implemented activities in contraceptive services during pregnancy and postpartum.

## Discussion

The findings show how a QIC supported by a register (SPR), and user involvement, may enable immigrant women to choose more effective contraceptive methods postpartum.

Few studies have included immigrants when seeking to improve contraceptive services in clinical practice even though immigrants often report less use of effective contraceptive methods [12, 13]. A previous study found that the quality of care for immigrant women improves when midwives regularly reflect together with other midwives upon the challenges regarding cultural diversity [37], supporting our findings.

In this study, midwives expressed that immigrant women were in greater need of support regarding reproductive decisions compared to native-born women. Previous studies also suggest that increased knowledge among HCPs helps strengthen reproductive autonomy among women [17, 18] which could be reflected in our findings of an increase in the proportion of immigrant women choosing a more effective contraceptive method.

Over the study period, the change in the proportion of immigrant women choosing a more effective method was seen early in the QIC. The reasons for this are not clear; however, it is possible that the fact that the change happened early in this QIC might be explained by the application of visual effectiveness tool [11] and double counselling sessions [5], had an immediate effect. Another possible explanation for that the effect happened early could be the due to the space for midwives to reflect and immediately focus on contraceptive services with colleagues from other MHCs, an intervention described to be effective in previous studies [33, 38].

Midwives also expressed the view that introducing new questions about contraception postpartum, and systematically recording this data in a register, prompted conversations about this, an effect, to our knowledge, not previously described. Feedback of stratified data from the SPR may also have increased midwives’ awareness of women’s choices of contraceptive methods in relation to immigrant status. Before the QIC, there was no collection of national data on women’s choice of contraception postpartum in the SPR, and thus it was not possible to systematically evaluate the effect of improvement activities. Hence, these findings are suggestive of the benefits described in previous studies of using a quality register to improve services [24].

### Methodological considerations

A major strength of this study is that the reported results appear associated with the use of a QIC with improvement activities designed to apply evidence-based knowledge in existing clinical practice for contraceptive services in the MHC.

It is not possible to isolate the specific improvement activity/ies that were associated with women’s choice of more effective contraception postpartum. In future research, there is a potential to distinguish between a varying influence of overlapping improvement activities by using the design of experiment methodology [37]. Even though, the QIC showed positive results, it was a small-scale study, and there is insufficient evidence as to how contraceptive services should be tailored to best help immigrant women choose a more effective method postpartum. A power calculation was not made, since at the outset this study was arranged as a pilot study. We included all women coming to the MHC clinics during the time of the QIC. To thoroughly evaluate the effectiveness of the QIC, a larger study is needed.

The study design did not include an evaluation of women’s actual use of contraception or their satisfaction with contraceptive counselling provided during the QIC. On the other hand, women gave their views on HCPs’ improvement activities, which strengthens the likelihood that services were responsive to women’s needs. Furthermore, following up on the sustainability of improvement activities after the QIC, we had to rely on midwives’ reports regarding changes in contraceptive services.

To our knowledge, throughout the study period, there were no changes in local or national policy regarding recommendations for contraception during and after pregnancy that may have influenced the study results. At the time of the QIC, an educational effort had recently been conducted at MHCs in Region Stockholm, including the MHCs participating in the study. The educational effort focused on improving attendance rates and developing postpartum services. However, it did not involve efforts to improve contraceptive services during pregnancy or postpartum visits.

The midwives took part in the interpretation of data regarding women’s choice of contraceptive methods during all LSs, as a form of interactive validation. Both qualitative and quantitative data in our study enabled triangulation, strengthening both the study’s construct and internal validity [31, 39]. Furthermore, we described how the QIC was led and presented quotes to show the relation between material and findings, thereby strengthening the study’s reliability [35].

### Implication for practice and future research

Our findings show the benefits of QIC and user feedback when developing contraceptive services in clinical practice. They also show the importance of having a register to monitor women’s contraceptive choices postpartum to evaluate how improvement activities may benefit immigrant women.

## Conclusions

A QIC supported by a register and user feedback was effective to help midwives to develop contraceptive services during the pregnancy and postpartum period, and may have facilitated the choice of more effective contraceptive methods postpartum by immigrant women.

## Data Availability

The dataset generated and/or analysed during the current study is not publicly available due to restrictions from the ethics review board but can be made available to qualified researchers upon request, after approval from the ethics board. EL should be contacted to request the data.

## Abbreviations

MHC: Maternal Health Clinic
QIC: Quality Improvement Collaborative
LS: Learning Sessions
UP: Unintended Pregnancy
HCPs: Health Care Professionals
LARC: Long-Acting Reversible Contraception
SARC: Short-Acting Reversible Contraception
SPR: Swedish Pregnancy Register
